# Clinical trials on pain lowering effect of ginger: A narrative review

**DOI:** 10.1002/ptr.6730

**Published:** 2020-05-20

**Authors:** Mariangela Rondanelli, Federica Fossari, Viviana Vecchio, Clara Gasparri, Gabriella Peroni, Daniele Spadaccini, Antonella Riva, Giovanna Petrangolini, Giancarlo Iannello, Mara Nichetti, Vittoria Infantino, Simone Perna

**Affiliations:** ^1^ IRCCS Mondino Foundation Pavia Italy; ^2^ Department of Public Health, Experimental and Forensic Medicine University of Pavia Pavia Italy; ^3^ Endocrinology and Nutrition Unit, Azienda di Servizi alla Persona “Istituto Santa Margherita” University of Pavia Pavia Italy; ^4^ Research and Development Unit Indena SpA Milan Italy; ^5^ General Management Azienda di Servizi alla Persona “Istituto Santa Margherita” Pavia Italy; ^6^ Department of Biomedical Science and Human Oncology University of Bari Aldo Moro Bari Italy; ^7^ Department of Biology, College of Science University of Bahrain Zallaq Bahrain

**Keywords:** chronic low back pain, dysmenorrhea, ginger, knee osteoarthritis, myalgia, pain

## Abstract

Ginger has a pain‐reducing effect and it can modulate pain through various mechanisms: inhibition of prostaglandins via the COX and LOX‐pathways, antioxidant activity, inibition of the transcription factor nf–kB, or acting as agonist of vanilloid nociceptor. This narrative review summarizes the last 10‐year of randomized controlled trials (RCTs), in which ginger was traditionally used as a pain reliever for dysmenorrhea, delayed onset muscle soreness (DOMS), osteoarthritis (AO), chronic low back pain (CLBP), and migraine. Regarding dysmenorrhea, six eligible studies suggest a promising effect of oral ginger. As concerned with DOMS, the four eligible RCTs suggested a reduction of inflammation after oral and topical ginger administration. Regarding knee AO, nine RCTs agree in stating that oral and topical use of ginger seems to be effective against pain, while other did not find significant differences. One RCT considered the use of ginger in migraine and suggested its beneficial activity. Finally, one RCT evaluated the effects of Swedish massage with aromatic ginger oil on CLBP demonstrated a reduction in pain. The use of ginger for its pain lowering effect is safe and promising, even though more studies are needed to create a consensus about the dosage of ginger useful for long‐term therapy.

## 1 INTRODUCTION

*Zingiber officinale* Roscoe (Zingiberaceae family), commonly known as ginger is a climbing perennial plant, indigenous to southeastern Asia. Ginger extract is a mixture of many biologically active constituents (Grzanna, Lindmark, & Frondoza, 2005). These compounds are numerous and vary depending on the place of origin and whether the rhizomes are fresh or dry (Ali, Blunden, Tanira, & Nemmar, 2008).

More than 400 chemical substances have been isolated and identified in ginger rhizomes extracts, and new ones are still being discovered (Charles, Garg, & Kumar, 2000; Jolad, Lantz, Solyom, & Chen, 2004; Ma, Jin, Yang, & Liu, 2004). At present, only a few of them have been evaluated for their pharmacological properties (Grzanna et al., 2005).

Carbohydrates, lipids, terpenes, and phenolic compounds are between the ones that already have been identified (Peter, 2016; Prasad & Tyagi, 2005). From the chemical point of view, isolated zingiber's principles are categorized into pungent and flavoring compounds. Pungent ones including gingerols, shogaols, zingerones, gingerdiols, gingerdione, and paradols are known to play a major role in various pharmacological actions (Jolad et al., 2004; Mashhadi et al., 2013; Peter, 2016; Prasad & Tyagi, 2005).

Patients suffering from diseases associated with chronic inflammation are turning to alternative compounds for relief of their symptoms or to take advantage of natural drug properties as prophylactic treatments. A complex interplay of *inflammatory* cells and a large range of chemical mediators are normally associated with the beginning of the inflammatory response, recruiting, and activating other immune cells to the site to subsequently solve it.

Several studies indicate that many different compounds in ginger are active in lowering chronic inflammatory diseases' symptoms:

- Inhibition of prostaglandins via COX and LOX pathways

The traditional use of ginger infusions to alleviate rheumatism and arthritis have pushed researchers to investigate the anti‐inflammatory pathways of secondary metabolites of the plant (Baliga et al., 2011; Zahedi, Fathiazad, Khaki, & Ahmadnejad, 2012). Some authors attributed 6‐gingerol's anti‐inflammatory activity to the inhibition of pro‐inflammatory cytokines and LPS‐activated macrophages antigen presentation (Setty & Sigal, 2005; Tripathi, Tripathi, Kashyap, & Singh, 2007).

Dugasani et al. demonstrated that shogaols and all gingerols inhibit NO production in LPS‐stimulated RAW 264.7 cells in a dose‐dependent manner (Dugasani et al., 2010). Moreover, with their experiments they showed that stimulation of RAW 264.7 cells with LPS (1 g/ml) for 24 hr induced a dramatic increase in PGE2 production, four times the basal level (Dugasani et al., 2010).

- Antioxidant activity on free radical scavenging cascade

*Zingiber officinale* active ingredients like gingerols, shogaols, zingerone, and so on exhibit antioxidant activity. Ginger inhibits an enzyme, namely xanthine oxidase, which is mainly involved in the generation of reactive oxygen species (ROS) (Ahmad et al., 2015).

- Inhibition of the transcription factor, nuclear NF‐kB

Grzanna and his group demonstrated for the first time that ginger inhibits the transcription factor nuclear NF‐kB (Grzanna et al., 2005). Nuclear NF‐kB is the principal regulator of pro‐inflammatory gene expression. Activated NF‐kB can be detected at sites of inflammation, and a link among NF‐kB activation, cytokine production, and inflammation is now generally accepted.

6‐gingerol displayed anti‐inflammatory activity by decreasing inducible NO synthase and TNF‐α expression through the suppression of I‐kBα phosphorylation, NF‐kB nuclear activation, and PKC‐α translocation. The compound was also found to control TLR‐mediated inflammatory responses. It inhibited NF‐kB activation and COX‐2 expression by inhibiting the LPS‐induced dimerization of TLR4 (Ahn, Lee, & Youn, 2009).

- Vanilloid nociceptor agonist

Vanilloid nociceptor agonists are known to be potent analgesics. The moderate pungency of ginger has been attributed to the mixture of gingerol derivatives in the oleoresin fraction of processed ginger. Gingerols possess the vanillyl moiety which is considered important for activation of the VR1 receptor expressed in nociceptive sensory neurones (Dedov et al., 2002).

Dedov reported that gingerols act as agonists at vanilloid receptors suggesting an additional mechanism by which ginger may reduce inflammatory pain (Dedov et al., 2002). This finding has added gingerols and zingerone to the list of vanilloid receptor agonists.

Moreover, the presence of VR1 receptors throughout the brainstem (Mezey et al., 2000), where the nausea center is located, may conceivably be associated in part with the common use of ginger as antiemetic medicine (Dedov et al., 2002).

In summary, current evidence in vitro and in animal models demonstrated that many different compounds of ginger have been shown to posses antioxidative and anti‐inflammatory activities that may be active in lowering chronic inflammatory disease symptoms, in particular pain.

However, human studies that were carried out to assess whether oral or topic ginger has a positive effect by reducing pain are not numerous; furthermore, in these studies different dosages and methods of administration were used, as well as disparate products' formulations and different study designs.

Given this background, the aim of this narrative review was to assess the state of the art randomized clinical trials on pain lowering effect of ginger, considering the pathologies in which ginger is traditionally used in order to control pain, such as: dysmenorrhea, delayed onset muscle soreness (DOMS), knee osteoarthritis, chronic low back pain (CLBP), and migraine.

## 2 MATERIALS AND METHODS

**FIGURE 1:**
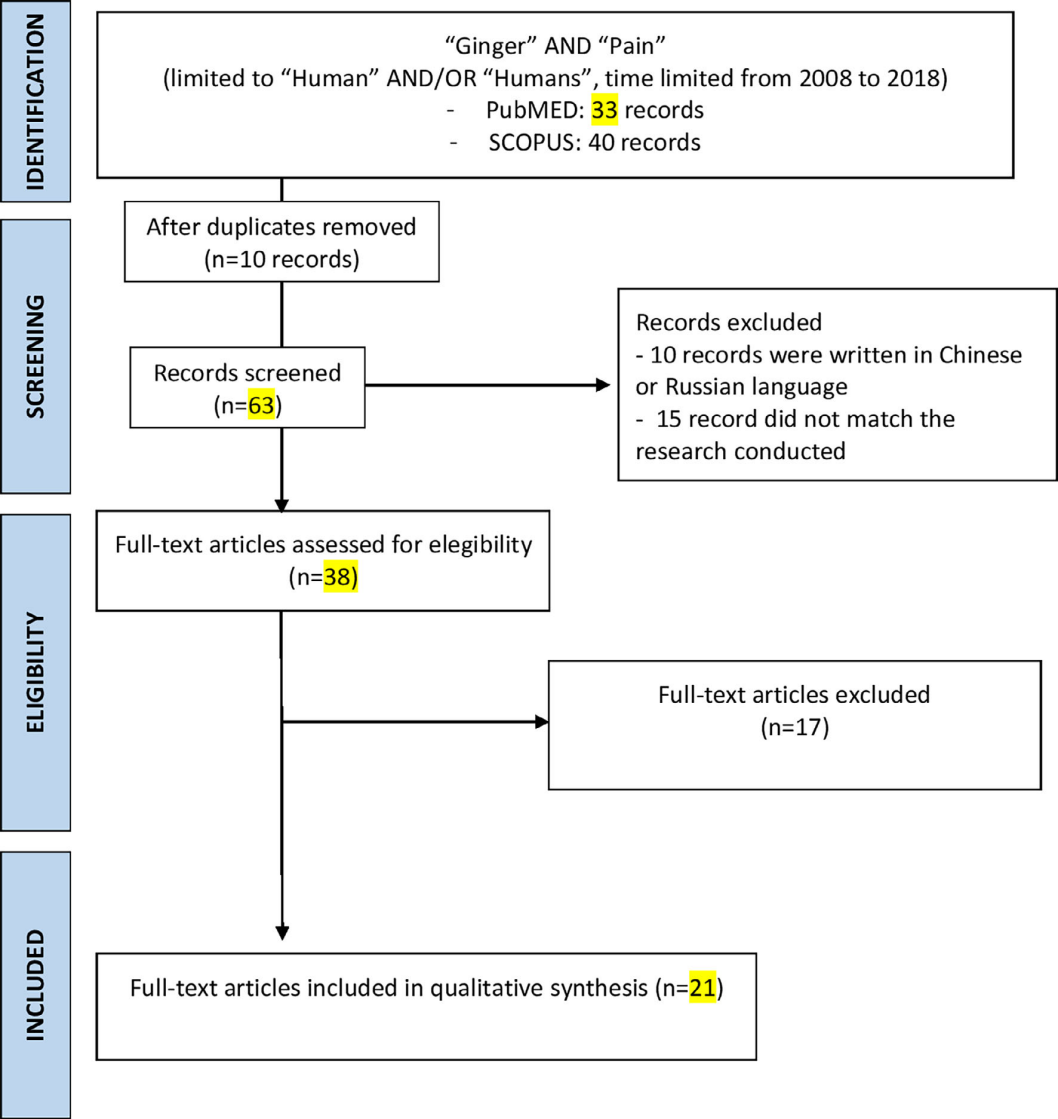
Flow chart of literature research [Colour figure can be viewed at wileyonlinelibrary.com]

This narrative review was written after a PubMed and SCOPUS research performed with these keywords: “Ginger,” “pain” with the use of Boolean AND operator to establish the logical relation between them. The research was conducted by four skilled operators from July to September 2018 and followed Egger's criteria (Egger, Smith, & Altman, 2001; Moher, Liberati, Tetzlaff, Altman,, & PRISMA Group, 2009). The research was time limited (from 2008 to 2018) and restricted to English and humans randomized controlled trial (RCT). The keywords were pain, ginger, primary dysmenorrhea, DOMS, knee pain, osteoarthritis (AO), CLBP, and migraine. They were combined with “AND” to search related articles. Furthermore, we selected trials with oral ginger used as a primary, sole or combined therapy and compared with a placebo or active treatment in diseases. The analysis was carried out in the form of a narrative review (Figure 1).

Standard datacoding tables were developed for extracting data from individual trials, key characteristics, and the level of evidence of each study (“Oxford Centre for Evidence‐based Medicine ‐ Levels of Evidence (March 2009)—CEBM,” 2009). The following data were extracted: participant characteristics, sample size, form and dosage of ginger, control group, assessment of adherence, outcome measures, methods for statistical analysis, study findings, and adverse events reported.

## 3 RESULTS AND DISCUSSION

### 3.1 Dysmenorrhea

This review summarizes evidence from six clinical trials (687 patients) evaluating the efficacy of oral ginger use for dysmenorrehea (Table 1). Among the six eligible studies, five were conducted in Iran (Jenabi, 2013; Kashefi et al., 2014; Ozgoli, Goli, & Simbar, 2009; Rahnama et al., 2012; Shirvani et al., 2017) and one in India (Halder, 2012). Participants were either college or high school students. Five of the six studies included only women with moderate to severe symptoms (Jenabi, 2013; Kashefi et al., 2014; Ozgoli, Goli, & Simbar, 2009; Rahnama et al., 2012). Five studies specified the inclusion of women with primary dysmenorrhea only, excluding women with secondary dysmenorrhea (Jenabi, 2013; Kashefi et al., 2014; Ozgoli, Goli, & Moattar, 2009; Rahnama et al., 2012; Shirvani et al., 2017); however, it is unclear how secondary dysmenorrhea was defined/diagnosed. Across the studies, the sample sizes of the ginger group ranged from *N* = 25 to *N* = 61. The daily dose of powdered ginger ranged from 750 to 2,000 mg. The most common duration and timing of ginger treatment was 3 days (the first 3 days of menstruation) (Halder, 2012; Jenabi, 2013; Ozgoli, Goli, & Simbar, 2009).

**TABLE 1:** Summary of articles about effect of ginger on dysmenorrhea

Author, year	Study design	Study population	Type of intervention	Results	Evidence level
Rahnama, Montazeri, Huseini, Kianbakht, & Naseri, 2012	Double blind, placebo‐controlled and parallel‐group study with balanced randomization [1:1] for the two groups.	one hundred twenty female students living in the dorms in Iran: age (>18), were randomly assigned to two equal groups: (a) ginger, (b) placebo	*Zingiber officinale* R. rhizomes. Five hundred milligram ginger powder for capsule and the others were placebo capsules, three times a day in two different treatment protocols. Both treatment protocols were given at monthly intervals.	Ginger may be an effective and safe therapy for relieving pain in women with primary dysmenorrhea if administered at the onset and 3 days earlier it needs revision.	Level IB
Shirvani, Motahari‐Tabari, & Alipour, 2017	Randomized clinical trial	One hundred twenty‐two female students living in the dorms in Iran: age (21.62 ± 2.0), were randomly allocated to two interventional groups: (a) ginger, (b) mefenamic groups.	Zingiber officinal roscoe. Two hundred fifty milligram capsule of ginger powder (Zintoma): in the mefenamic group every 8 hr, and in the ginger group every 6 hr from the onset of menstruation until pain relief lasted 2 cycles.	No significant difference in pain severity was found between ginger and mefenamic.No significant difference in pain duration was found between ginger and mefenamic acid.	Level IB
Kashefi, Khajehei, Tabatabaeichehr, Alavinia, & Asili, 2014	A placebo‐controlled randomized trial	One hundred fifty high school students (15–18 age) in Iran, were randomly assigned to three study groups. (a) zinc sulfate (*n* = 48), (b) ginger (*n* = 56), (c) placebo (*n* = 46)	Ginger: The relative capsules were filled with 250 mg ginger powder. The capsules was filled with 220 mg zinc sulfate, and the placebo capsules were filled with lactose.	The severity of pain was significantly different between, before, and after the intervention in both the ginger and the zinc sulfate groups.	Level IB
				Compared with the placebo receiving group, participants receiving ginger and zinc sulfate reported more alleviation of pain during the intervention.	
				The severity of dysmenorrhea was not significantly different among the three groups before the intervention.	
Halder, 2012	Clinical trial	Seventy‐five nursing students in India. Participants were divided into three groups: experimental group 1, experimental group 2 and control group again by lottery method, 25 in each group.	The first experimental group was administered Jacobson's progressive muscle relaxation exercise once a day; the second experimental group was administered ginger powder 1 g per dose twice a day with warm water after meal.	Main outcome measures were the severity of selected symptoms of dysmenorrhoea, which were analysed using MANOVA.	Level IIA
			Pre‐test post‐test control group design was selected.	In treating symptoms of dysmenorrhoea, ginger powder has efficacy superior to progressive muscle relaxation.	
Jenabi, 2013	Randomized clinical trial	Seventy college students in Iran, were allocated to two groups: (a) Ginger group (*n* = 35), (b) Placebo group (*n* = 34), aged 21.33 ± 1.16/ 21.54 ± 1.78 years	Ginger versus placebo, both in capsule form for 3 days in first menstruation cycles.	In the ginger group, 29 subjects reported an improvement in nausea symptoms, compared with 16 in the placebo group. Ginger is effective in minimising the pain severity in primary dysmenorrhoea.	Level IB
			Five hundred milligram of it was filled in each capsule.		
Ozgoli, Goli, & Moattar, 2009	Double‐blind comparative clinical trial	One hundred fifty college students (>18) in Iran living in the dorm, were allocated to three groups: (a) ginger, (b) mefenamic acid, (c) ibuprofen.	Ginger powder versus placebo: The ginger group took 250 mg capsules of ginger rhizome powder four times a day for 3 days from the start of their menstrual period. Members of the other groups received 250 mg mefenamic acid or 400 mg ibuprofen capsules, respectively, on the same protocol.	No significant difference in pain severity was found between ginger, ibuprofen, and mefenamic acid.	Level IIA

Dysmenorrhea is characterized by low abdominal or pelvic pain occurring before or during menstruation (Morrow & Naumburg, 2009). Better management of dysmenorrhea may not only improve women's quality of life, but also reduce their risk of developing future pain (Berkley & McAllister, 2011; Vincent et al., 2011). Dysmenorrhea is conventionally treated with nonsteroidal anti‐inflammatory drugs (NSAIDs) or oral contraceptive pills (OCPs) (Dawood, 2006), the efficacy of which are supported by research evidence (Wong, Farquhar, Roberts, & Proctor, 2009). However, NSAIDs and OCPs have limitations: some women with dysmenorrhea do not respond to NSAIDs or OCPs (with an estimated failure rate of >15% for NSAIDs) (Dawood, 2006); some cannot use these medications because of contraindications or adverse effects; some prefer not to use any medications. Therefore, investigation of complementary alternative treatments for dysmenorrhea is warranted. Ginger is one of the most commonly used natural products among women with dysmenorrhea. The exact mechanism of action of ginger in pain relief remains to be elucidated; however, some evidence suggests that the constituents of ginger have anti‐inflammatory and analgesic properties (Ali et al., 2008). Furthermore, preclinical research shows that ginger suppresses the synthesis of prostaglandin (through inhibition of cyclooxygenase) and leukotrienes, which are involved in dysmenorrhea pathogenesis (Dawood, 2006; Committee on Herbal Medicinal Products (HMPC), 2013).

The available data suggest a promising pattern of oral ginger (750 to 2,000 mg for the first 3 days of menstruation) as a potentially effective treatment for pain in dysmenorrhea. All RCTs agree in demonstrating that ginger is more effective for pain relief than placebo, and no significant difference was found between ginger and NSAIDs (Halder, 2012; Jenabi, 2013; Kashefi et al., 2014; Ozgoli, Goli, & Moattar, 2009; Rahnama et al., 2012). These findings, however, need to be interpreted with caution due to the small number of studies, poor methodological quality, and high heterogeneity across the trials. Moreover, all of the included trials were conducted in Asia.

### 3.2 Delayed onset muscle soreness

This review summarizes evidence from four randomized clinical trials (194 subjects) evaluating the efficacy of oral or topic ginger use for DOMS (Table 2). Three RCT suggested an effective reduction of inflammation due to exercise‐induced muscle damage after daily consumption of 2 g of raw and heat‐treated ginger (Black et al., 2010; Manimmanakorn et al., 2016). A useful alternative seems to be the topical administration of *Zingiber cassumunar* in 14% concentration (Manimmanakorn et al., 2016). Finally, 4 g of ginger supplementation is the suggested dose to be used to accelerate recovery of muscle strength following intense exercise (Matsumura et al., 2015).

**TABLE 2:** Summary of articles about effect of ginger on DOMS

Author, year	Study design	Study population	Type of intervention	Results	Evidence level
Matsumura, Zavorsky, & Smoliga, 2015	Double‐ blind, randomized placebo‐controlled trial.	Twenty Non‐weight trained partecipants allocated in two groups. (a) Intervention group: *n* = 10 (5F, 5 M); aged 32 ± 9 years. (b) Placebo group: *N* = 10 (5F, 5 M); 27 ± 5 years.	Zingiber officinale roscoe (4 g) once daily for 5 days.	Four gram of ginger supplementation may be used to accelerate recovery of muscle strength following intense exercise but does not influence indicators of muscle damage (DOMS).	Level IB
Manimmanakorn et al., 2016	Double‐ blind, randomized placebo‐controlled trial.	Seventy‐five healthy untrained volunteers (47F, 28 M), aged 18–60 years, allocated in three groups. (a) 14% Plai cream: *n* = 25 (15F, 10 M); aged 28.7 ± 13.7 years. (b) 7% Plai cream: *n* = 25 (16F, 9 M); aged 31.3 ± 16.7 years. (c) Placebo group: *n* = 25 (16F, 9 M); aged 26.2 ± 12.0 years.	*Zingiber cassumunar*: 2 g of the cream (strips of 5‐cm long) were rubbed into the quadriceps muscles for 5 min immediately following the exercise and every 8 hr thereafter for 7 days in all groups.	Using 14% Plai cream over a 7‐day period substantially reduced muscle soreness symptoms compared to 7% Plai cream or a placebo cream.	Level IB
Black & O'Connor, 2008	Double blind crossover design	Twenty‐five participants (15F, 10 M) aged 23.2 ± 4.2 years.	Six capsules, for a total of 2 g of ground ginger or 2 g of flour (placebo), administered 30 min before cycling on an ergometer at an intensity of 60% of VO_2peak,_ enough to stimulate mild to moderate quadreceps muscle pain.	Ginger exhibited no hypoalgesic effect on quadriceps pain intensity compared with placebo.	Level IIA
Black, Herring, Hurley, & O'Connor, 2010	Double‐ blind, randomized placebo‐controlled trial	Study 1: *N* = 34 allocated in two groups: (a) Raw ginger: *n* = 17 (14F, 3 M), aged 21.1 ± 0.7 years. (b) Placebo: *n* = 17 (14F, 3 M), aged 20.9 ± 0.6 years.	Six capsules, for a total of 2 g of raw or heated ginger or 2 g of placebo, all administered within 1 min, before performing 18 eccentric actions of the nondominant elbow flexors at an intensity of 120% of their concentric 1‐RM.	Raw and heat‐treated ginger resulted in similar pain reductions 24 hr after eccentric exercise compared to placebo.	Level IB
		Study 2: *N* = 40 allocated in two groups: (a) Heated ginger: *n* = 20 (13F, 7 M), aged 20.6 ± 0.6 years. (b) Placebo: *n* = 17 (13F, 7 M), aged 21.4 ± 0.8 years.			

DOMS indicated by muscle pain and tenderness typically occurs after a strenuous workout or undertaking unaccustomed exercise (Gulick & Kimura, 1996). The underlying causes of DOMS are related to exercise‐induced muscle damage, including sarcomere disruption, and the ensuing secondary inflammatory response^,^(Gleeson et al., 1995; Warren, Hayes, Lowe, Prior, & Armstrong, 1993). Inflammatory processes stimulate prostaglandin E2 release which sensitizes type III and IV pain afferents, and leukotrienes to attract neutrophils, which produce free radicals that further exacerbate muscle cell damage (Connolly, Sayeres, & McHugh, 2003). DOMS after eccentric exercise may result in reduction of muscle performance of athletes (Cheung, Hume, & Maxwell, 2003). Numerous methods to prevent and reduce DOMS have been suggested, including stretching exercises, massage, and nutritional supplementation. Non‐steroidal anti‐inflammatory drugs (NSAIDs) have been used in an attempt to reduce DOMS by reducing inflammation and pain and improving function.

Ginger and several of its constituents inhibit activity of COX‐1 and COX‐2, block leukotriene synthesis, and block the production of interleukins and tumor necrosis factor alpha in activated macrophages (Black et al., 2010). Thus, these antiinflammatory actions that may help reduce inflammation, such as exercise‐induced muscle damage, are recognized as a product of participating in unfamiliar or strenuous physical activity.

Given that ginger has antinflammatory and analgesic properties, it follows that it may be used to reduce the damage and consequent DOMS following high‐intensity exercise. (Black et al., 2010; Black & O'Connor, 2008).

### 3.3 Knee osteoarthritis

This review summarizes evidence from nine randomized clinical trials (964 patients) evaluating the efficacy of oral or topical ginger, sole or in combination with other botanicals, use for knee OA (Table 3). The three RCTs that considered the efficacy of oral ginger (powder supplementation of 1 g/day) on pain in knee OA demonstrated that efficacy of ginger on knee pain seemed not to have a unique position. Two studies suggested that the use of ginger in subjects with knee pain could decrease pain (Mozaffari‐Khosravi et al., 2016; Naderi et al., 2016) while other did not find significant differences (Naderi et al., 2016; Niempoog et al., 2012). The two RCT that assessed the efficacy of the topical use of ginger considered an aromatic essential oil (1% *Zingiber officinale* and 0.5% Citrus sinesis) (Yip & Tam, 2008) and 4% ginger gel (Niempoog et al., 2012) and agree in stating that the topical use of ginger seems to have potential as an alternative method for short‐term knee pain relief. Finally, all the four studies that considered the ginger supplementation in combination with other botanicals agree in demonstrating that these combinations are effective in order to decrease knee pain (Chopra et al., 2013; Drozdov et al., 2012; Nieman et al., 2013).

**TABLE 3:** Summary of articles about effect of ginger on knee AO

Author, year	Study design	Study population	Type of intervention	Results	Evidence level
Zahmatkash & Vafaeenasab, 2011	A double‐blind randomized controlled trial study.	Ninety‐two participates with the mean age of 52.2 (+/− 12.4) years, allocated to two groups: (a) Ginger, (b) Salicylate	Ginger versus salicylate: 2 g of topical ointment for three times a day, applied with massage for 1 min, for 6 weeks, twice a day. Treatment group applied herbal ointment and control group used salicylate ointment.	It seems that using this herbal combination is clinically effective for patients suffering from knee AO in order to decrease their pain, morning stiffness and limited motion; its effect is comparable with salicylate ointment. The severity of pain were measured using Visula analog pain scale.	Level IB
Niempoog, Siriarchavatana, & Kajsongkram, 2012	A double‐blind, randomized, controlled trial.	Fifty participates, male and female, allocated to two groups: (a) Treatment (Plygersic) gel, (b) Diclofenac gel	Zingiber officinale Zingiber cassumunar versus diclofenac: The combination of 4% ginger and plai extract in a gel (Plygersic gel) as compared with a 1% solution of diclofenac sodium gel.	Both Plygersic gel and diclofenac gel could significantly improve knee joint pain, symptoms, daily activities, sports activities and quality of life measured by KOOS following 6 weeks of treatment. In the ANOVA fo repeated measures, there were no differences in the results between the Plygersic and diclofenac gel groups.	Level IB
			Gels were applied as 1 g solution for four times a day for 2 months	The efficacy of the drugs was monitored by using KOOS (knee injury and AO outcome score) score.	
Mozaffari‐Khosravi, Naderi, Dehghan, Nadjarzadeh, & Fallah Huseini, 2016	A randomized double‐blind placebo‐controlled trial	One hundred twenty participates, aged 50–70 years, allocated to two groups: (a) Ginger group (GG), (b) Placebo group (PG).	GG group: 500 mg of ginger powder, while PG participants received capsules filled with 500 mg starch.	At baseline proinflammatory cytokine (TNF‐alfa and IL‐1 g) concentrations did not differs in the groups. At 3 months, both cytokines decreased in the GG relative to the PG group.	Level IB
			Partecipants were treated twice daily for 3 months.	The results of this study indicate that ginger supplementation may have a promising benefits for knee AO.	
Naderi, Mozaffari‐Khosravi, Dehghan, Nadjarzadeh, & Huseini, 2016	Double blind randomized placebo controlled clinical trial.	One hundred twenty patients with moderate painful knee OA, aged 50–70 years, Male and female, allocated to two groups: (a) Ginger, (b) Placebo	Two capsules of 500 mg per day for 3 months.	At the beginning of the study there were no significant difference between the two groups in terms of C‐reactive protein and nitric oxide. After 12 weeks the concentration of these markers declined more in the ginger group. Ginger supplementation at this dosage can reduce inflammatory markers in patients with knee AO	Level IB
			Capsules of ginger and placebo look the same and contains ginger and starch powder, respectly		
Niempoog et al., 2012	A double‐blind randomized controlled trial.	Sixty patients, allocated to two groups: (a) Treatment group (*n* = 30), (b) Placebo group, aged 48.88 years (male); 49.09 years (female), allocated to two groups: (a) Ginger, (b) Placebo	Zingiber officinale (rhizome) versus placebo: 500 mg powered ginger in one capsule, twice a day for 2 months vs the same looking capsule of placebo twice a day.	The present study showed that 1 g per day of powdered ginger could not relieve joint pain and improve symptoms and the quality of life during 8 weeks of treatment of AO of the knee compared with the placebo.	Level IB
Nieman et al., 2013	Placebo‐controlled, randomized, double‐blind clinical trial.	One hundred men and women, ages 50–75 years with a history of join pain >3 months, allocated to two groups: (a) Instaflex, (b) Placebo	InstaflexTM is a joint pain supplement containing glucosamine sulfate, methylsufonlylmethane (MSM), white willow bark extract (15% salicin), ginger root concentrate, boswella serrata extract (65% boswellic acid), turmeric root extract, cayenne, and hyaluronic acid (4.0 mg).	Results from this support the use of the Instaflex dietary supplement in alleviating joint pain severity in middle‐aged and older adults, with mitigation of difficulty performing daily activities most apparent in subjects with knee pain.	Level IB
			Treatment: 1 gel capsule for three times a day for 8 weeks for INSTAFLEX and placebo.		
Chopra et al., 2013	Randomized, double‐blind, parallel‐efficacy, four‐arm, multicentre equivalence drug trial.	Four hundred forty eligible patients suffering from symptomatic knee OA were enrolled and monitored as per protocol. Age > 55, four groups: Ayurvedic groups (two shunthi‐guduchi formulations, SGCG and SGC), vs glucosamine sulfate or celecoxib	Ayurvedic formulations SGCG and SGC (without Boswelia Serrata) are extracts of Tinospora cordifolia (73.33 mg), Zingiber officinale (33.33 mg), Emblica officinalis (166.66 mg), *Boswellia serrata* (100 mg). Two capsules for three times a day with palin water after a meal or snack.	Ayurvedic drugs were found equivalent to oral glucosamine sulfate and celecoxib in reducing knee pain and improving knee function.	Level IB
			Glucosamine sulphate (2 g daily) and celecoxib (200 mg daily) for 24 weeks.		
Yip & Tam, 2008	Double‐blind, placebo‐controlled clinical trial	Of the 59 participants enrolled in this study, 53 (89.8%) participants completed both post 1‐week and 4‐week follow‐up. The majority of the 53 participants were women (79%) and the mean age was 73.59 years old (S.D. = 5.42 years). Their mean knee joint pain history was 9.71 years (S.D. = 7.05 years). Partecipants were allocated to groups: (a) Intervention group (IG), (b) Placebo control group (PG), (c) Control group (CG).	Ginger oil versus orange oil: Ginger essential oil (1% ginger and 0.5% *Citrus sinensis* oil in olive oil as the base lubricant) and conventional treatment. 0.5% orange essential oil (*Citrus aurantium*).	The intervention group reported a reduction in knee pain rating (at the 4‐week follow‐up period. No significant differences were reported in mean change in stiffness intensity for betweengroup comparison at thepost 1‐week follow‐up or post 4‐week follow‐up.	Level IB
			Sessions of 30–35 min of aroma massage on both lower imbs, six times witin 2–3 weeks.		
Drozdov, Kim, Tkachenko, & Varvanina, 2012	Randomized clinical trial.	Forty‐three patients (35F, 8 M), aged 55.1–3.1 years, with an average disease duration of 7.1–1.3 years, were allocated to two groups: (a) Ginger, (b) Diclofenac	The ginger group received a specific ginger combination daily (340 mg EV.EXT 35 Zingiber officinalis extract) for 4 weeks. The diclofenac group received 100 mg diclofenac daily for the same period. Both groups also received 1,000 mg glucosamine daily.	The ginger group showed a slight but significantly decreased upper SODA pain intensity by the 28th day of treatment with *p* = .05. SODA dyspepsia did not change significantly during the treatment and the dyspepsia index remains the same with *p* = .6. EGDS showed significantly increased levels of PGE1, PGE2, and PGF2a in the stomach mucosa. The diclofenac group showed increased SODA pain and dyspepsia values with a corresponding significant decrease of stomach mucosa prostaglandins and general negative stomach mucosa degeneration.	Level IB
		The ginger group with 21 patients (17F, 4 M) and. The diclofenac group (positive control) of 22 patients (18F, 4 M)			

Osteoarthritis Osteoarthritis (OA) is a joint disease characterized by degeneration of cartilage, pain, inflammation, impaired mobility, and dysfunction, especially in older populations (Heidari, 2011).

Current treatment for OA is palliative and is focused on pain relief and improving mobility, including a combination of nonpharmacologic and pharmacologic measures. However, when these therapeutic treatments fail to improve symptoms, a variety of surgical interventions can be used (Haghighi, Tavalaei, & Owlia, 2006). With respect to some commonly‐used treatments, such as NSAIDs, there is increasing concern that long‐term consumption may cause gastrointestinal bleeding and cardiovascular risks, mainly hypertension and thrombotic events (Dingle, 1999; Mamdani, 2005). Thus, an interest in research has been conducted to find care treatments that have negligible adverse effects while offering significant improvements in the symptoms (Zakeri et al., 2011).

Ginger is thought to have anti‐inflammatory effects and may modulate the concentration and activity of inflammatory mediators in OA (Ahmad et al., 2015).

Only one RCT, that considered the efficacy of oral ginger (powder supplementation of 1 g/day) on pain in knee OA, did not find significant changes (Niempoog et al., 2012). These difference in the results between the study by Niempoog et al. and the study by Mozaffari‐Khosravi H, although the dosage used was the same (1 g/day), could be due to numerous reasons: the type of administration (non‐encapsulated powder vs. encapsulated powder), the duration of treatment (2 vs. 3 months), the parameters studied (symptoms and daily activities vs. inflammatory cytokines), number of patients evaluated (30 vs. 120 subjects) (Mozaffari‐Khosravi et al., 2016; Niempoog et al., 2012).

### 3.4 Chronic low back pain

As shown in Table 4, one RCT was sourced using topical ginger. No RCT studies were found regarding oral ginger supplementation and pain in patients with low back pain.

**TABLE 4:** Summary of articles about effect of ginger on migraine and low back pain

Author, year	Study design	Study population	Type of intervention	Results	Evidence level
Sritoomma, Moyle, Cooke, & O’Dwyer, 2014	Randomized controlled trial	One hundred sixty‐four patients were screened; 140 were eligible, and randomized to two groups: (a) Swedish massage with aromatic ginger oil (SMGO) (*n* = 70), (b) TTM (*n* = 70). Female and male, aged 60 years.	Aromatic ginger oil used for Swedish massage in SMGO group contains 10 ml of jojoba oil and 2% of aromatic ginger oil for massaging. Traditional Thai Total sample massage (TTM).	Both SMGO and TTM led to significant improvements in pain intensity (*p* < .05) and disability (*p* < .05) across the period of assessments, indicating immediate, short‐ and long‐ term effectiveness. SMGO was more effective than TTM in reducing pain (*p* = .04) and improving disability at short‐ and long‐term assessments (*p* = .04).	Level IB
Cady et al., 2011	A double‐blind placebo‐controlled pilot study	Sixty patients, female and male, aged 12–60 years with migraine, were randomized allocated 3:1 into two groups: (a) feverfew/ginger, (b) placebo.	Forty‐five subjects for a total of 208 attcks of migraine over 1 month, Were treated with feverfew/ginger: 2 units dose applications. If any headache pain persists at 1 hr, a second treatment of 2 units could be administred.	Sublingual formulation of feverfew/ginger appears safe and effective as a first‐line abortive treatment for a population of migraineurs who frequently experience mild headache prior to the onset of moderate to severe headache.	Level IIB
			Fifteen subjects of the placebo group treated 58 attacks with a sublingual placebo preparation.		

CLBP is defined as a chronic condition of lower back pain lasting for at least 3 months or longer (Andersson, 1999; Bogduk, 2004). Non‐pharmacologic interventions for CLBP are recommended when patients do not show improvement with standard treatment (Deyo, Mirza, & Martin, 2006).

Ginger has been used as an anti‐inflammatory and anti‐rheumatic for musculoskeletal pain (Altman & Marcussen, 2001; Therkleson, 2010). Although CLBP predominantly affects older people, only one study has specifically investigated the effects of Swedish massage with aromatic ginger oil in order to evaluate if improve the level of disability.

The objective of Sritoomma et al. was to investigate the effects of Swedish massage with aromatic ginger oil (SMGO) on CLBP and disability in older adults compared with traditional Thai massage (TTM). This study demonstrated that SMGO was more effective than TTM in reducing pain and improving disability at short‐ and long‐term assessments. It's important to note that there might be a lack of methodology in this study, because the difference might be due to the type of massage not to the use of ginger. Moreover, it is not clear if the benefit of low back pain is due to the massage and ability of the operator itself or the medical principle of ginger drug in the aromatic oil.

### 3.5 Migraine

One single RCT (Cady et al., 2011) on humans could be considered as the first effort to elucidate the use of ginger in this frequently disabling disease that is present in a large slice of the adult population. This multi‐center RCT on feverfew/ginger use seems to suggest an effective treatment to migraine.

Migraine is a complex neurological disease characterized by episodic periods of disabling physiological dysfunction typically recurring over decades of an individual's lifetime (Cady, Schreiber, & Farmer, 2004).

Treatment needs for migraine vary considerably from patient to patient and indeed, from attack to attack for the same patient. Pharmacologically, many over the counter and prescription products are effective on treatment in acute migraine (Monteith & Goadsby, 2011). Commonly employed acute treatments approved for migraine can be classified as over the counter products, such as acetaminophen/aspirin/caffeine combination products and non‐steroidal anti‐inflammatory medications (Wenzel, Sarvis, & Krause, 2003), and prescription products, such as triptans and non‐steroidal anti‐inflammatories (Ng‐Mak, Hu, Chen, & Ma, 2008). Various opioid and butalbital‐containing analgesics are also commonly prescribed, but most do not have Food and Drug Administration approval for migraine, and are generally avoided by physicians because of their propensity to produce medication overuse headache (Bigal, Rapoport, Sheftell, Tepper, & Lipton, 2004; Snow, Weiss, Wall, & Mottur‐Pilson, 2002).

The main limitation of this review is represented by searching just two databases and only English literature.

## 4 CONCLUSION

Current evidence in vitro and in animal models demonstrated that many different compounds of ginger have been shown to posses antioxidative and anti‐inflammatory activities that may be active in lowering chronic inflammatory disease symptoms, in particular, pain. This pain‐reducing effect of ginger has been modulated through various mechanisms: inibition of prostaglandins via the COX and LOX‐pathways, antioxidant activity, inibition of the transcription factor nf–kB, or acting as agonist of vanilloid nociceptor.

However, human studies that were carried out to assess whether oral or topic ginger has a positive effect by reducing pain are not numerous; furthermore, in these studies different dosages and methods of administration were used, as well as different study designs.

This narrative review summarizes the last 10‐year of RCT, in which ginger was traditionally used as a pain reliever for dysmenorrhea, DOMS, knee AO, CLBP, and migraine.

Regarding dysmenorrhea, six eligible studies suggest a promising effect of oral ginger. As concerns DOMS, the four eligible RCTs suggested a reduction of inflammation after oral and topical ginger administration. Regarding knee AO, eight RCTs agree in stating that oral and topical use of ginger seems to be effective against pain, while others did not find significant differences. One RCT considered the use of ginger in migraine and suggested its beneficial activity. Finally, one RCT evaluated the effects of Swedish massage with aromatic ginger oil on CLBP demonstrated a reduction in pain.

The most of the included trials were conducted in Asia. Pharmacogenetics and outcome expectancy regarding ginger intervention could differ across cultures and ethnicities, and therefore, it is necessary to confirm the promising effects in the worldwide population.

In conclusion, the use of ginger for its pain lowering effect is safe and promising, even if more studies are needed to create a consensus about the amount of ginger useful for long‐term therapy. The positive health benefits need to be interpreted with caution due to the small number of studies, poor methodological quality, and high heterogeneity across the trials. Therefore, new studies, possibly multi‐center RCT in different continents, with an adequate number of patients and with standardized ginger formulations, are necessary to confirm the results of this review.

## CONFLICT OF INTEREST

The authors declare no conflicts of interest.
